# Bilateral ocular manifestations of Sturge-Weber syndrome: a rare case report

**DOI:** 10.22336/rjo.2024.32

**Published:** 2024

**Authors:** Aparajita Chaudhary, Shivani Garg

**Affiliations:** * Department of Ophthalmology, Moti Lal Nehru Medical College, Prayagraj, India

**Keywords:** Sturge-Weber syndrome, port wine stain, anterior segment optical coherence tomography

## Abstract

The rare neurocutaneous condition known as Sturge-Weber syndrome (SWS) is characterized by leptomeninges, or angiomas affecting the face, eyes, and brain. We report a newly diagnosed case that came to our institute complaining of a diminution of vision BE that had been going on for the past 1 year. Upon examination, the patient exhibited bluish discoloration of the sclera, an increase in the size of the cornea, and the characteristic port wine stain (PWS) on the face.

Intraocular pressure BE was 30 mmHg with an applanation tonometer. The cup disc ratio on fundoscopy was 0.9 RE and 0.8 LE with characteristic glaucomatous disc changes BE. The child was treated with antiglaucoma medications.

**Abbreviations:** SWS = Sturge-Weber syndrome, PWS = Port wine stain, CNS = Central nervous system, CT = Computed Tomography, IOP = Intraocular pressure, OCT = Optical coherence tomography, RE = Right eye, LE = Left eye, BE = Both eyes, ASOCT = Anterior segment optical coherence tomography

## Introduction

SWS belongs to a group of disorders known as phakomatoses. These are congenital tumors that are usually hamartomas [**1**]. It is also labeled as encephalotrigeminal angiomatosis. It encompasses three classical manifestations facial angiomatosis or nevus flammeus, ipsilateral leptomeningeal hemangioma, and ocular hemangioma [**2**]. Bilateral involvement is rare.

For confirmatory diagnosis, there should be at least two manifestations of the classical triad (PWS, leptomeningeal angioma, and eye abnormalities). SWS can present many clinical symptoms in various situations, either with or without ocular involvement [**3**,**4**].

The most difficult ocular anomaly associated with SWS is glaucoma. Glaucoma typically presents unilaterally and is primarily detected during infancy, but late-onset glaucoma can occasionally arise during adulthood in a few cases [**5**].

Cases of unilateral glaucoma have not been reported in the literature so far. To the best of our knowledge, this is the first case of bilateral glaucoma with bilateral PWS reported.

## Case report

**Fig. 1:**
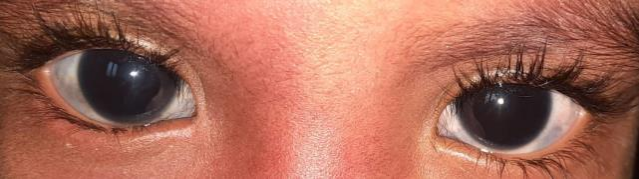
Bluish discoloration of the sclera in both eyes

A 4-year-old female child diagnosed with SWS was referred to our hospital for evaluation of a large size of cornea. The patient complained of severe diminution of vision BE for the last 1 year. It was gradual in onset, and progressive in nature. On examination, reddish discoloration on her face and body was observed and has been present since birth (**Fig. 1**).

No history of convulsions, trauma, or any other previous ocular surgical procedure in the eyes was present.

No similar complaints or history in her siblings and other family members were noted. Other systemic examinations were unremarkable.

**Fig. 2:**
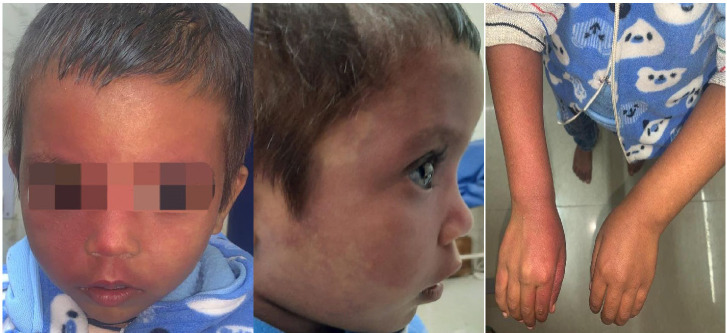
Bilateral port wine stain on face and body

On ocular examination, the following were observed: visual acuity of Finger count - 2 mt in both eyes, no improvement on best corrected visual acuity, perception of light to be present, and projection of rays accurate in all quadrants. A bluish discoloration of the sclera was present in BE (**Fig. 2**). The corneal diameter of 13 mm vertically and 14 mm horizontally was measured with the caliper in BE. The cornea was clear, and transparent, deep anterior chamber, and pupillary reaction sluggish BE. Axial length was found to be 23.73 mm RE and 24.56 mm LE. Intraocular pressure in both eyes was 30 mmHg with the applanation tonometer. Gonioscopic examination revealed a closed angle in all four quadrants in BE. Fundus examination with 90 D Slit-lamp Biomicroscopy revealed a cup disc ratio of approximately 0.9 RE and 0.8 LE. Thinning of neuroretinal rim BE was observed. 

**Fig. 3:**
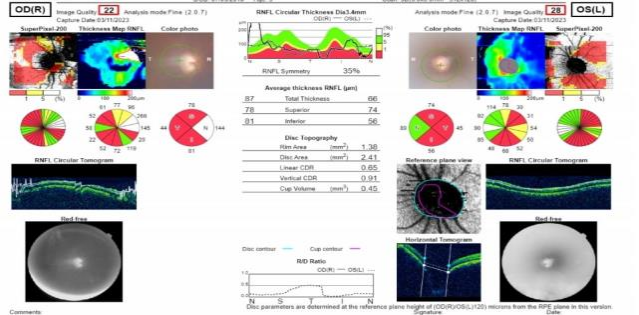
Retinal Nerve Fibre Layer thinning of both eyes in OCT Optic Nerve Head

On ASOCT, central corneal thickness RE was 591 microns, and LE was 571 microns. OCT of the optic nerve head and retinal nerve fiber layer of BE confirmed cupping and all neural parameters seemed to be compromised (**Fig. 3**).

**Fig. 4:**
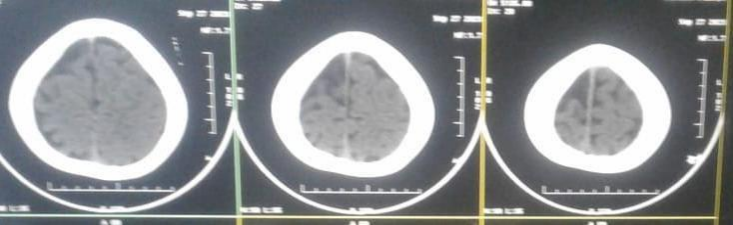
Subtle subcortical calcification in the left temporo-occipital and right parieto-occipital region, without any volume loss on CT brain

CT Brain revealed subtle subcortical calcification in the left temporo-occipital and right parieto-occipital region, without any volume loss (**Fig. 4**). Clinical history and the above findings suggested Sturge-Weber syndrome. Despite the CT scan findings, no neurological signs or symptoms were observed.

She was given initial medical treatment on her first visit. It included Dorzolamide eyedrops three times a day, prostaglandin analog one drop at night, lubricating eye drops four times a day BE, and Tab methylcobalamin once a day. On follow-up, intraocular pressure was 20 mmHg. The patient was asked to come for follow-up at regular intervals and the attendant was counselled for surgery in the future if required.

## Discussion

Complete SWS is defined as having both face and CNS angiomas and incomplete when only one area is affected without the other. The Roach Scale is used for classification, as follows [**6**,**7**]:

Type I - Both facial and leptomeningeal angiomas; may have glaucoma; 

Type II - Facial angioma alone (no CNS involvement); may have glaucoma; 

Type III - Isolated leptomeningeal angioma; usually no glaucoma.

The case reported was of Complete Type I Sturge-Weber Syndrome. 

**Table 1:** Clinical manifestations of SWS and manifestations observed in our case

No	Clinical features	Incidence (%)	Present case
1.	Epilepsy	80	-
2.	Port wine stain	76	+
3.	Abnormal radiographic findings	63	+
4.	Mental retardation	54	-
5.	Oral Manifestations	38	-
6.	Hemiparesis	37	-
7.	Ocular manifestations	37	+

Nema N et al. (2014) stated that bilateral glaucoma is observed in about 45% of patients with bilateral SWS [**8**]. INAN (1999) noted that 87–90% of the port wine stains are limited to the right side of the face in these circumstances. Lesion extension beyond the median is present in 50% of patients, and 33% are affected on both sides [**9**]. In this case, the child additionally demonstrated a port wine stain on both sides of the face.

According to Waelchli et al., PWS distribution may follow the embryonic vasculature distribution of the face, rather than along the trigeminal nerve [**10**].

Facial PWSs can be successfully treated by laser. The laser should be applied superficially to avoid potential problems such as cerebral venous outflow decrease through PWS vessels. Choroidal vessel dilatation, exudative retinal detachment, and intraocular pressure increase could be caused by the problems that may arise. As a result, when treating PWS, deep photocoagulation and debulking surgery need to be avoided [**11**].

Glaucoma management aims to reduce IOP and prevent further damage to the visual field and optic nerve. Since it is rare, topical antiglaucoma medications are more difficult to treat in the congenital form, but they are the first line of treatment in the late-onset variant [**12**].

However, in SWS, the surgical success rate is the lowest among secondary glaucoma, since surgical failure, uncontrolled IOP, and low vision outcomes have been frequently reported [**13**]. The development of this ocular complication still represents the worst prognostic factor for vision loss in SWS patients, despite the availability of medical and surgical approaches to treat both forms of glaucoma [**5**].

## Conclusion

SWS is a chronic illness, even though patients may not have symptoms throughout their early years. Glaucoma is the most frequent ocular complication. To prevent the loss of visual function, surgery is often necessary to achieve long-term control of intraocular pressure (IOP) due to its early development and poor response to conventional medical therapy. Furthermore, the surgical success rate for secondary glaucoma in SWS is the lowest because of angle abnormality. The appearance of clinical aspects determines how the SWS is treated.

Due to the wide spectrum of clinical manifestations of SWS, management of the condition requires multidisciplinary teams, including pediatricians, neurologists, ophthalmologists, cosmetologists, physiotherapists, radiologists, and dentists.

Prompt diagnosis is essential to prevent further issues. Patients and their parents must receive professional psychiatric counseling. 

**Conflict of Interest Statement**

The authors state no conflict of interest.

**Informed Consent and Human and Animal Rights Statement**

All appropriate patient consent forms were obtained. The patient’s attendant gave her consent for her child’s images and other clinical information to be reported in the journal. The patient’s attendant understands that her name and initials will not be published and due efforts will be made to conceal her identity, but anonymity cannot be guaranteed. 

**Authorization for the use of human subjects**

Ethical approval: The research related to human use complies with all the relevant national regulations, and institutional policies, as per the tenets of the Helsinki Declaration, and has been approved by the review board of Moti Lal Nehru Medical College, Prayagraj, India (IEC/MLNMC/2024/No.61 & 25/06/2024).

**Acknowledgments**

None.

**Sources of Funding**

None.

**Disclosures**

None.
